# Modern Light-Cured Restorative Composites as Luting Agents: The Effect of Preheating on Conversion and Film Thickness

**DOI:** 10.3390/ma18163721

**Published:** 2025-08-08

**Authors:** Maria Dimitriadi, Aikaterini Petropoulou, Ioannis Papathanasiou, Spiros Zinelis, George Eliades

**Affiliations:** 1 Department of Biomaterials, School of Dentistry, National and Kapodistrian University of Athens, 115 27 Athens, Greece; szinelis@dent.uoa.gr (S.Z.); geliad@dent.uoa.gr (G.E.); 2 Department of Prosthodontics, School of Dentistry, National and Kapodistrian University of Athens, 115 27 Athens, Greece; apetrop@dent.uoa.gr (A.P.); johnpapatha@dent.uoa.gr (I.P.)

**Keywords:** preheated composites, luting agents, degree of conversion, film thickness, FTIR spectroscopy

## Abstract

The aim of this study was to evaluate (a) the degree of conversion (DC%), (b) film thickness, and (c) the effect of film thickness on DC% in modern light-cured resin composite restoratives [Filtek Universal (F), Clearfil Majesty ES 2 Universal (M), Tetric EvoCeram (T) and Viscalor (V)] used for luting composite onlays before/after preheating. For (a), the luting composites placed at 150 μm film thickness under the onlays (4 mm thickness, 2.9% transmittance) were light-cured for 120 s (3 × 40 s top, buccal, lingual sites) before and after preheating (54 °C/5 min-F,M,T and 65 °C/30 s-V). The DC% was measured at central, middle and side locations along the median in-length axis by ATR-FTIR spectroscopy. Specimens polymerized without onlays (40 s, top) served as controls. For (b), film thickness was measured employing a modified ISO 4049 standard (37 °C plate temperature, 5 N load) before and after preheating, using a dual-cured resin luting agent as control. For (c), onlays were luted with preheated T at 150 and 350 μm film thickness and light-cured for 2 × (3 × 40) s and 3 × (3 × 40) s, employing directly irradiated specimens (60 s, 120 s) as controls. For (a), significant differences were found in F and T before and after preheating. Before preheating, significant differences were registered between F–T, F–M, F–V and V–T, whereas after they were registered between F–M, F–T and F–V. All these values were significantly lower than the controls. For (b), significantly lower film thickness was recorded after preheating (−16.1–−33.3%, highest in V), with a ranking of F, M > V > T (before) and F, M > T, V (after). All values were significantly higher than the control. For (c), increased exposure improved DC% in the greater spacer group, with the controls providing superior values. It can be concluded that the use of modern highly filled composites as luting agents for low translucency onlays may result in suboptimal polymerization and film thickness, warranting caution.

## 1. Introduction

Indirect restorations of ceramic and resin composite materials are extensively used in the rehabilitation of damaged teeth, where minimally invasive techniques are deemed as the most effective approach [1,2]. These restorations are typically bonded using resinous luting agents, employing micromechanical and adhesive strategies. For an acceptable and durable bonded interface, several mechanical, structural, chemical, biological and aesthetic criteria should be met [1,2,3], with viscosity and handling characteristics being equally significant. Currently, a variety of resinous luting agents are available, classified according to their activation mechanism (light-, self-, dual-cured) and adhesive capacity (adhesive, self-adhesive), and mediated by conditioners, primers and adhesive agents. To fulfill the major requirement of low film thickness, these materials are reinforced with a lower volumetric filler loading than traditional restorative composites, resulting in inferior properties such as elastic modulus, fracture toughness, flexural strength, solubility, wear resistance and color stability [4,5,6,7,8].

The use of highly filled restorative composites for luting purposes faces the problem of high viscosity and the resultant great film thickness [9]. Several techniques have been introduced to reduce film thickness, like restoration seating assisted by a sonicating probe or preheating [10]. Preheating has been shown to substantially reduce viscosity of direct resin composite restoratives and film thickness, while for sonication the results are controversial [11,12]. Generally, for luting applications an optimal film thickness (<120 μm) is advised [13,14,15]. Nevertheless, there are reports for marginal discrepancies in indirect restorations varying between 100 and 315 μm [14].

An additional issue raised for light-cured luting materials is the curing capacity because the intensity of the activating light is strongly attenuated, especially in thick restorations made of materials with a low translucency parameter [6,16,17]. The degree of C=C bond conversion (DC) of luting materials is important for durable bonding. A high DC enhances the mechanical and chemical characteristics of the luting agent, improving the load-bearing capacity, stress distribution and fracture resistance of the restoration, thereby providing supplementary support to the underlying tooth structure [1].

Preheating has been reported to improve the DC of light-cured restoratives used for luting purposes [6,18,19]. However, in several studies, an insignificant effect of preheating on DC and a rapid temperature reduction after removal from the heating device has been documented [4,8,9,20,21]. This controversy has been further complicated by reports supporting different reaction mechanisms to preheating among various composites [14].

Recently, new categories of direct resin restorative composites have been introduced, such as the group-shaded materials for easier shade selection, the thermoviscous, offering the advantage of direct application of preheated material on-site, and materials with addition–fragmentation monomers to compensate shrinkage. The aim of the present study was (a) to evaluate the DC of these materials used for luting indirect low translucency composite onlays before and after preheating, (b) to assess the film thickness of these materials before and after preheating, and (c) to evaluate the effect of film thickness on DC. The null hypotheses were as follows: (a) there are no significant differences in the DC of the materials before and after preheating, (b) there are no significant differences in film thickness before and after preheating, and (c) the film thickness does not affect DC.

## 2. Materials and Methods

The materials tested are listed in Table 1. The materials represent most modern groups of light-cured resin composite restoratives including conventional bisphenol-A monomer adducts, BPA-free aromatic monomers, addition–fragmentation monomers and thermoviscous formulations. To be used as luting agents, all the materials were preheated at 54 °C for 5 min (Caps Warmer, Voco), except for the thermoviscous V, where a dedicated preheating gun was used (VisCalor Dispenser, Voco, 65 °C/30 s).

**Figure 1:**
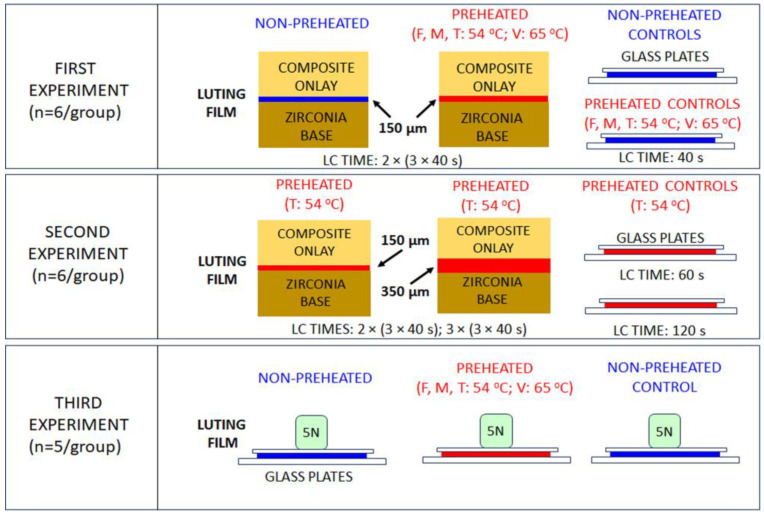
The experimental design of the study.

The experimental procedure employed is summarized in Figure 1.

### 2.1. Curing Capacity

#### 2.1.1. Curing Capacity of Luting Light-Cured Composites Under Onlays at Standard Thickness

CAD/CAM zirconia frameworks (3Y-TZP, A3 shade 12 mm mesio-distal length, 4 mm bucco-lingual width, 10 mm height) with 150 μm spacers were fabricated as a background simulating an onlay preparation, which received resin composite onlays (Crea.lign, Bredent, Chesterfield, UK, A3 shade, 12 mm length, 4 mm width, 4 mm height). The total transmittance of the composite specimens was 2.9% at 468 nm, as measured with a 50 mm diameter integrated sphere (RSA-PE-20, PerkinElmer, Norwalk, CT, USA) attached to a UV-Vis spectrophotometer (Lambda 35, PerkinElmer). The zirconia and onlay intaglio surfaces were highly polished to avoid mechanical retention of the luting agent. Each luting composite was preheated as above, applied to the framework surface, which was kept at 37 °C in a dry heat oven, and pressed with the onlay to obtain good contact with the spacers; excess was removed with a sharp instrument. Immediately after, the composite was light-cured from lateral (bucco-lingually) and top sites for 120 s (40 s each side) using a LED curing unit (Radii Plus, SDI, Bayswater, Australia) emitting 1180 mW/cm^2^ light intensity, as measured with a curing radiometer (Bluephase II, Ivoclar Vivadent). Another series of specimens were prepared without preheating (room temperature 24 °C). Finally, specimens prepared by pressing the luting composites against the frameworks with transparent microscopic glass slides covered with cellulose matrix strips and directly irradiated for 40 s were used as controls. For each experimental condition, six specimens were prepared per material and stored for 10 min at 37 °C (dark/dry).

#### 2.1.2. Curing Capacity of Luting Light-Cured Composite Under Onlays at Different Thicknesses

To evaluate the effect of luting agent thickness on the curing capacity, zirconia frameworks with 150 and 350 μm spacers were fabricated, onlays were bonded with one conventional preheated composite (T), and irradiated as follows: 2 × 120 s (40 s per side) and 3 × 120 s (40 s per side), employing the same curing unit. The specimens (*n* = 6 per thickness and irradiation mode) were stored as before. Another series of specimens were prepared by pressing the luting composite against the framework with a transparent microscopic glass slide covered with a cellulose matrix strip. After excess removal, the specimens were directly irradiated for 60 s and 120 s. The specimens (*n* = 6 per irradiation mode) were stored as previously described. This group served as a control of directly irradiated specimens.

#### 2.1.3. Measurement of the Curing Capacity

**Figure 2:**
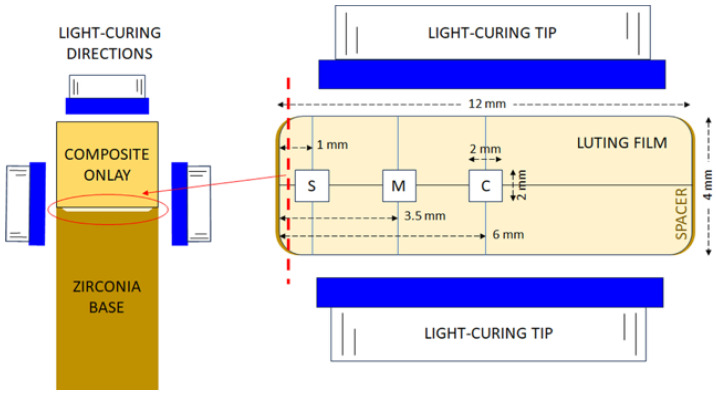
Details of specimen preparation and the location of the luting film areas analyzed. Left: vertical cross-sectional view of the set-up. Right: top view of the specimen dimensions relative to the light-curing tip placed for lateral irradiation (C: central, M: middle, S: side locations). The dotted line of the right image corresponds to the cycled cross-sectional view of the left image.

For 2.1.1 testing conditions, the onlays were removed from the zirconia framework and the degree of C=C conversion (DC%) of the luting composites facing the framework were analyzed at the central part (designated as C, 6 mm inner to the restoration width edge), the middle part (M, 3.5 mm inner to the width edge) and the side part (designated as S, 1 mm inner to the restoration margins) along the middle length line of each specimen (Figure 2). For the directly irradiated specimens, the central region was analyzed. Measurements were performed with attenuated total reflection FTIR spectroscopy (ATR–FTIR), employing an ATR accessory (Golden Gate, Specac, Oprington, Kent, UK), with a single-reflection diamond element (2 × 2 mm) and ZnSe lenses, attached to an FTIR spectrometer (Spectrum GX, PerkinElmer, Buckinghamshire, Bacon, UK). Spectra were acquired under the following conditions: 4000–650 cm^−1^ wavenumber range, 4 cm^−1^ resolution, 20 scans co-addition, and ≈2 μm depth of analysis at 1000 cm^−1^. Spectra of uncured composite pastes served as controls. For the DC% measurements, the aliphatic C=C stretching vibrations at 1635 cm^−1^ were chosen as the analytical band (AN), whereas the aromatic C..C stretching vibrations at 1605 cm^−1^ (for M, T, V) or the N–H bending vibrations at 1540 cm^−1^ (for F) were selected as the reference bands (RF). Quantification was performed according to the following equation: DC% = 100 × [1 − (ApAN × AmRF/AmAN × ApRF)], where A is the net peak absorbance height of set (p) and unset (m) materials at analytical (AN) and reference (RF) bands, respectively.

For 2.1.2 testing conditions, measurements were performed at C-designated locations, whereas, for the directly irradiated groups, measurements were performed at the central regions. DC% was estimated for T as above.

### 2.2. Film Thickness

A modification of the ISO 4049:2019 (7.5) [22] was employed to measure the film thickness of the materials under loading conditions used during seating of prosthodontic restorations. Briefly, the thickness of two glass plates (upper, lower > 5 mm) in contact was measured with a digital micrometer (Digimatic Micrometer 293-832, Kanagawa, Japan, ±1 μm resolution). The plates were placed in a dry heat oven at 37 °C to simulate intraoral temperature. A standardized composite volume (0.05 mL) measured with a modified micro-fine syringe (sectioned tip base) was placed on the lower plate, covered with the upper plate and vertically loaded at the center of the top plate with 5 N (≈510 g) for 180 s. Then, the load was released, the specimens were irradiated for 80 s with the same curing unit as before, the total thickness was measured again and the net plate thickness was subtracted to calculate the specimen film thickness. The procedure was repeated (*n* = 5) for preheated specimens and for specimens loaded at room temperature (24 °C). For F, M and T, preheating (54 °C) was performed on preloaded modified micro-fine syringes, whereas for V (65 °C) the 1/4 of the 0.25 g compule volume (≈0.05 mL) was applied directly from the heat-dispensing gun. The dual-cured adhesive luting agent, (E) applied at 24 °C and light-cured as above, was used as a control.

### 2.3. Statistical Analysis

The normality and equal variance of the mean value distributions was assessed by Shapiro–Wilk and Brown–Forsythe tests, respectively. For the first experiment, a three-way ANOVA was used to evaluate the effects of the independent variables “material”, “condition” and “location” on DC% under the onlays, whereas one-way ANOVA was used for DC% comparisons of each material per heating mode vs. the native controls. Comparisons between the two controls per material (preheated vs. non-preheated) were performed by *t*-tests. For the second experiment, a two-way ANOVA (independent variables: “spacer size” and “exposure time”) was executed, followed by one-way ANOVA of the controls vs. the spacer groups at each condition. Finally, for the third experiment, one-way ANOVA was employed to assess differences among the materials and their controls per heating mode, while individual differences between the two heating modes per material were evaluated by *t*-tests. The ANOVA analyses were followed by multiple comparison tests (Holm–Sidak), to isolate group differences. All analyses were performed employing the Sigma Plot v.15 software (Systat Software Inc., San Jose, CA, USA) at a 95 % confidence level (α = 0.05).

## 3. Results

### 3.1. Curing Capacity

#### 3.1.1. Curing Capacity of Luting Light-Cured Composites Under Onlays at Standard Thickness

**Figure 3:**
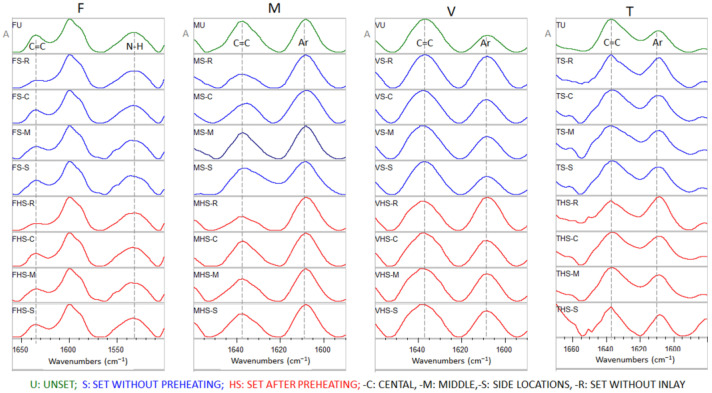
Representative ATR–FTIR spectra of the materials tested per condition and location with the bands used for DC% measurements (absorbance scale). F: Filtek Universal, M: Clearfil Majesty ES-2 Universal, V: VisCalor, T: Tetric EvoCeram.

Representative ATR–FTIR spectra of unset and set materials obtained at different locations and temperature conditions with the analytical and reference bands used for DC% measurements are illustrated in Figure 3. The quantitative results per material are presented in Table 2. The mean DC% values of the specimens irradiated under the onlays subjected to three-way ANOVA (independent variables: “material”, “condition” and “location”) showed no statistically significant difference among the different levels of “location” and the effect of differences in “material” and “condition” (*p* = 0.194). A statistically significant interaction was found between “material”–“location” (*p* = 0.020), while there was an insignificant interaction between “condition”–“location” (*p* = 0.635) and “material”–“condition” (*p* = 0.425). In the non-preheated mode, significant differences were found between F–T, F–M, F–V (*p* < 0.001) and V–T (*p* = 0.03), whereas in the preheated mode the significant differences were limited between F–M (*p* = 0.008), F–T (*p* = 0.032) and F–V (*p* = 0.046). For the variable “condition”, significant differences were found only in F (*p* = 0.024) and T (*p* = 0.036) between preheated and non-preheated groups. To compare the DC% per material vs. the individual controls (40 s direct light exposure) under the two heating conditions, one-way ANOVA (M, T, V) or one-way ANOVA on Ranks (normality failure for F) were used. For all materials, the DC% values were significantly higher in the controls. No significant differences were found between the controls per material (non-preheated vs. preheated) when tested separately (*t*-test).

#### 3.1.2. Curing Capacity of Luting Light-Cured Composite Under Onlays at Different Thicknesses

**Figure 4:**
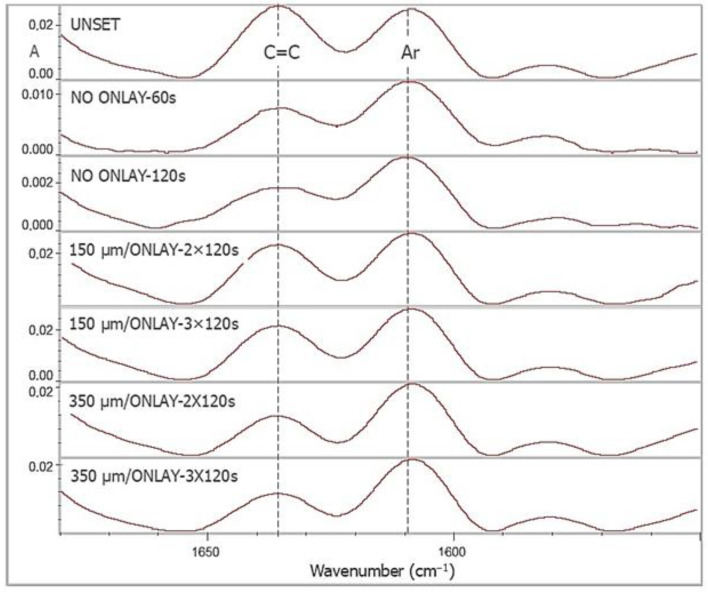
ATR–FTIR spectra of T film, unset/set without onlays and set under onlays at 150 and 350 μm thickness, with the bands used for DC% measurements (absorbance scale).

ATR–FTIR spectra obtained for DC% measurements of T, along with the controls, are illustrated in Figure 4. The results of DC% are summarized in Table 3. A two-way ANOVA (independent variables: “spacer size” and “exposure time”) showed significant differences for both variables (*p* ≤ 0.001), without significant interactions (*p* = 0.317). Increased exposure improved DC%, with the greater spacer providing higher values for both the exposure times tested. For comparisons with the controls, one-way ANOVA was used, revealing a superior performance of controls over the spacer groups regardless of the exposure time (*p* ≤ 0.001), except for the 60 s control with the 350 μm spacer group irradiated for 2 × 120 s (*p* > 0.05). The analysis showed that the 120 s irradiated control was superior to the 60 s analog (*t*-test, *p* < 0.001).

### 3.2. Film Thickness

The results of film thickness measurements for the materials tested are presented in Table 4. A two-way ANOVA (general linear model, independent variables: “materials” and “conditions”, controls: E and non-preheated groups) demonstrated significantly higher values in E and the preheated groups (*p* < 0.001). The percentage reduction ranged from 16.1 to 19.5% for the materials preheated at 54 °C (F, M, T), and 33.3% for the thermoviscous (V, 65 °C). The ranking of the film thickness values before preheating was F, M > V > T, whereas after preheating F, M > T, V (*p* < 0.05). All composite restoratives demonstrated significantly thicker films in comparison with E, irrespective of the heat treatment mode.

## 4. Discussion

The experimental setup used in the first experiment simulated the use of light-curing composite restoratives for luting-extensive onlay restorations, with full chromatic characterization (enamel/dentin A3 shades). Since onlay restorations are indicated for teeth with extensive hard tissue loss, the limitation of 2 mm thickness of indirect semi-transparent restorations for adequate light transmission [23] cannot be met. For comparison among luting materials, a standardized luting thickness of 150 μm was used. This size was selected to (a) comply with the design directions given for the cement space in most currently available resin composites (mainly CAD/CAM) of complex design, with the actual sizes exceeding the acceptable limit of 120 μm [14,24,25,26], and (b) to ensure the same cross-sectional area is exposed to lateral irradiation, irrespectively of the luting agent viscosity and seating pressure applied. The specific light-curing unit used has four LED chips tuned in the blue wavelength range (450 nm peak emission), with an effective emission area of 0.443 cm^2^ (Ø: 0.75 cm in circular analog), and a radiant emittance exceeding 1000 mW/cm^2^ only at the central 0.5 cm diameter, although the nominal value reported is 1346 mW/cm^2^ (420–495 nm range) [27]. Since similar problems appear in many LED curing units, three testing locations (C, M, S) were selected for the DC measurements to take into consideration the beam profile deviations [28]. Moreover, measurements were taken at the central in-length axis of the specimens representing the most distal point to the irradiated margins, where light attenuation upon lateral irradiation is the highest. In the absence of information about the temperature compatibility of M and T, preheating of restoratives F, M and T was performed at 54 °C, a temperature within the acceptable range for significant viscosity reduction [28]. A 5 min preheating period was chosen in compliance with most studies [28]. For the thermoviscous V, the compules used with the dedicated dispenser delivered on-site preheated material at 65 °C, which has been considered as an advantage, avoiding the rapid temperature drop during transferring from the heater [8,20,21]. All DC% measurements were performed after 10 min storage at 37 °C under dry conditions. Although post-curing time is known to enhance DC, the differences measured range from 2 to 18% dependent on the initial exposure time and irradiance [29,30]. In the present study, conditions ensuring exposure to high photon energy (40 s exp, 1180 mW/cm^2^ irradiance) were used for high initial conversion, which, in turn, may diminish the extent of post-curing effects on DC. For F, which demonstrated a complex aromatic peak (central peak at 1600 cm^−1^ with a shoulder at 1590 cm^−1^), the N–H peak was selected as a reference band for better quantification.

Preheating had a positive effect on F and T, which agrees with the concept of different reaction mechanisms to preheating among composites [14]. Although V was preheated at a 17% higher temperature (65 °C vs. 54 °C), no positive effects were encountered at any location. It should be mentioned that in groups directly exposed to high intensity irradiation (controls), preheating improved DC% in F at significantly higher values than M, T and V, although insignificant differences existed in the non-preheated groups. Addition–fragmentation chain transfer monomers (AFM), as the one contained in F, have been reported to reduce photoreactivity and DC [31,32]. A possible explanation is the absence of conventional bisphenol–dimethacrylate monomers in F, which hinder conversion, and the presence of highly reactive and flexible aliphatic dimethacrylates, such as DDMA and DUDMA [33], which may counterbalance any hampering effect of AFM and respond better to the increased thermal mobility induced upon preheating. The low DC% values of the restoratives under the onlays in comparison with the controls, despite the prolonged irradiation time of the former, are mainly ascribed to the attenuation of the activating light due to the reduced cross-sectional area available for direct exposure of the luting material from lateral margins and the strong light absorption by the onlay material. Such low values may create problems in the integrity and biocompatibility of the restorations, since it has long been documented that DC controls an array of mechanical, chemical and biological properties of resin composites [34].

Although the effect of restoration thickness and translucency on the DC of light-cured luting agents has been investigated [35,36,37], the effect of film thickness under relatively opaque structures has received limited attention. The results of the second experiment clearly showed that thick luting layers demonstrated improved DC%, apparently due to the increased cross-sectional area available for lateral irradiation simulating buccolingual exposure. Measurements were performed only with T, since this material represents a typical well-documented composite, without the specific developments introduced in other materials. The DC% values of T in the 150 μm group for total exposure times of 280 s and 360 s demonstrated insignificant differences and ranged between 32 and 38%. These values were similar to the corresponding material in the first experiment under 120 s irradiation, and significantly less than the 60 s exposed controls. Therefore, it can be concluded that extended irradiation cannot improve conversion at the 150 μm space size due to limited light penetration capacity. However, when the spacer size increased to 350 μm, the DC significantly increased, reaching the level of the 60 s control group. This indicates that film thickness is the principal factor for high bulk DC of light-cured composite luting agents under onlays with minimal light transmission, provided that lateral irradiation is feasible.

In the absence of a consensus, the increased film thickness of light-cured restorative composites has been considered as clinically acceptable due to their improved mechanical properties in comparison to resinous luting agents [7]. However, materials with thickness exceeding 80 μm under 30 N load have been characterized as unsuitable for preheated luting agent applications [38]. Therefore, important questions are raised about the proper seating of indirect restorations when thick composite luting films are used. Nevertheless, in clinical studies of inlay restorations with 90 μm predetermined cement space, marginal gap sizes between 144 and 280 μm have been registered [24], which doubts the rationale of the criteria set for material acceptance as above.

The thickness of the luting materials, apart from viscosity, greatly depends on the seating forces applied. Although it has been suggested that the use of preheated composites for luting inlays, onlays and overlays does not prevent seating accuracy [39], this was claimed to apply only for some composites [38]. Official assessment of film thickness for resinous luting agents involves a standardized methodology under 150 N load [22]. However, this load has negligible clinical relevance as a seating load, especially for complex and delicate restorations, such as glass–ceramic or composite inlays, onlays, overlays and endocrowns. A wide range of seating loads, other than the official one, has been proposed in many studies (0.1–75 N) [3,38,39,40,41] in compliance with specific testing requirements. Moreover, in other studies, undefined loads have been applied by finger pressure, which may vary between 12 and 67 N [42]. In the current study, a 5 N load was used for the film thickness test, as it has been found to induce the highest reinforcement in biaxial flexural strength of ceramics [43]. To better simulate oral temperature, the testing plates were kept at 37 °C in a dry heat oven, where the entire experiment was performed.

The film thickness measurements confirmed the significant viscosity reduction after preheating of composite restoratives [44]. The lowest film thickness in the non-preheated group was observed in T, a highly filled composite with 80–83 w% total filler content but with the lowest inorganic filler content of all. The same material, after preheating at 54 °C, showed similar performance with V, the highest filled material with inorganic fillers preheated at 65 °C. This supports previous claims that the effect of preheating is material dependent [14], since variations in monomer chemistry, filler content, size, shape and filler–resin interfacial interactions affect the rheological properties of composites [45,46]. Despite the temperature-induced shear thinning of the light-cured composite restoratives under the modified film thickness testing conditions used, the values of all materials were significantly greater than the flowable adhesive dual-curing luting agent used as a control, without preheating. This finding is in accordance with previous studies on the advantages of flowable materials over conventional and packable composites, irrespective of the preheating status, even under the high loading advised in the ISO 4049 specification [47]. The film thickness of the adhesive dual-curing luting agent used was reported to be ≤50 μm, apparently in line with the relevant specification, while the value recorded under the current testing conditions was twice as large. Nonetheless, for brittle materials (such as ceramics or highly filled composites [48]), film thickness values from 50 to 200 μm have been found to increase the bending strength of the critical material–cement interface [49]. Consequently, the restrictions on film thickness for luting agents should be carefully reviewed considering the current experimental evidence on the marginal and internal adaptation of indirect inlay/onlay restorations.

Based on the results of the present study, the first null hypothesis should be accepted, except for F and T. In all cases, the DC% of the composite luting agents cured under the onlays were lower than the directly irradiated controls, although irradiated at a fraction of the total energy of the latter. The second null hypothesis should be rejected, since preheating significantly reduced film thickness in all products. However, the values recorded were significantly higher than the adhesive dual-cured luting agent used as control. Finally, the third null hypothesis should be rejected, since great film thickness enhanced DC mainly due to increased photon flux density from lateral irradiation, reaching the control values. Nevertheless, interfacial gaps of this size contradict the design principles of indirect restorations regarding cavity adaptation. The complex interactions documented in the present study establish the need for further research on this topic. An issue would be to examine whether the low DC of light-cured restorative composites used as luting agents under inlays/onlays with reduced translucency parameter can be increased by using dual-cured adhesives, as documented for some direct light-cured restoratives [50,51]. Until methods ensuring an adequate DC are secured, the use of light-cured direct restorative materials for such applications should be approached with skepticism.

## 5. Conclusions

Under the experimental conditions of the present study, the following conclusions can be reached:

Preheating had a positive effect only on the conversion of F and T. The directly irradiated controls showed significantly higher conversion, although light-cured at a fraction of the energy provided for the bonded specimens.

Preheating significantly reduced film thickness in all materials. However, no material provided values similar with the dual-cured luting agent control tested at room temperature.

The size of film thickness is implicated with the conversion capacity under the opaque onlays; the greater size enhances conversion due to increased lateral photon flux.

## Figures and Tables

**Table 1:** The materials used in this study.

Materials	Composition *	Manufacturer
**INDIRECT ONLAY MATERIAL**		
Crea.lign dentine A3	*Resin:* Mixture of DUDMA isomers, BGDMA. *Filler:* Nanosized opalescent ceramic filler (50 w%).	Bredent, Chesterfield, UK
**DIRECT LIGHT-CURED COMPOSITE RESTORATIVES USED AS LUTING AGENTS**		
3M Filtek Universal A2 (F)	*Resin:* AUDMA, AFM, DUDMA, DDMA, catalysts, stabilizers, water. *Filler:* Non-aggregated silica (20 nm), zirconia (4–11 nm), aggregated zirconia/silica clusters, aggregated YbF_3_ (100 nm), (76.5 w%, 58.4 v% inorganic filler).	3M, St. PaulMN, USA
Clearfil Majestry ES-2 Universal (M)	*Resin*: BisGMA, hydrophobic aliphatic dimethacrylate, catalysts, stabilizers, pigments. *Filler:* Silanated Ba-glass filler, pre-polymerized organic filler, (66 w%).	Kuraray Noritake, Okuyama, Japan
Tetric EvoCeram A3 (T)	*Resin:* UDMA, BisGMA, BisEMA, catalysts, stabilizers, pigments. *Filler:* Ba-glass, mixed oxide, YbF_3_ and prepolymer additives (82–83 w%, 48.5 w% inorganics, 34 w% prepolymers, mean size of inorganic fillers: 550 nm, range 40 nm–3 μm).	Ivoclar Vivadent Schaan, Lichtenstein
VisCalor A2 (V)	*Resin:* BisGMA, TCDDMA, initiators, stabilizers, pigments. *Filler:* BaAlB-silicate glass, silica (83 w%).	Voco GmbH, Cuxhaven, Germany
**DUAL-CURED ADHESIVE RESIN LUTING AGENT**		
Variolink Esthetic DC (E)	*Resin:* UDMA, GDMA, DDMA, AUDMA *Filler:* YF_3_, spheroid mixed oxide (60–68 w%, 38 v%, mean size 0.1 μm, range 0.04–0.2 μm).	Ivoclar Vivadent Schaan, Lichtenstein

* According to manufacturers’ information. AFM: addition–fragmentation monomer; AUDMA: aromatic urethane dimethacrylate; BisEMA: ethoxylated bisphenol glycidyl dimethacrylate; BisGMA: bisphenol glycidyl dimethacrylate; BGDMA: butylene glycol dimethacrylate; DDMA: 1,12 dodecane dimethacrylate; DUDMA: Diurethane dimethacrylate; GDMA: glycerol dimethacrylate; TCDDMA: tricyclodecane dimethacrylate; UDMA: urethane dimethacrylate.

**Table 2:** Results of DC% measurements per material, condition and location (means and standard deviations). R: reference without onlay. S, M, C: side, middle and central locations. F: Filtek Universal, M: Clearfil Majesty ES-2 Universal, V: VisCalor, T: Tetric EvoCeram.

Material	Non-Preheated				Preheated			
	Location							
	R	S	M	C	R	S	M	C
**F**	50.94 (2.05) a,A,1	38.03 (3.85) a,B,1	37.03 (2.67) a,B,1	41.17 (3.86) a,Β,1	60.01 (6.97) a,A,2	40.98 (4.55) a,B,1	38.9 (2.8) a,B,1	40.88 (3.1) a,B,1
**M**	48.46 (3.23) a,A,1	34.9 (2.95) b,B,1	32.53 (0.74) a,B,1	35.4 (2.31) b,B,1	45.63 (5.64) b,A,1	33.57 (1.35) b,B,2	35.3 (2.03) b,B,2	34.27 (1.55) b,B,1
**T**	44.98 (1.73) b,A,1	27.44 (4.31) c,B,1	33.83 (3.27) a,B,1	33.38 (2.31) b,B,1	48.91 (8.51) b,A,1	35.8 (2.19) b,B,2	35.2 (2.88) b,B,1	35.7 (2.91) b,B,1
**V**	47.14 (5.67) a,A,1	32.68 (2.91) b,B,1	35.26 (3.54) a,B,1	35.47 (2.75) b,B,1	50.44 (3.95) b,A,1	31.83 (2.02) c,B,1	33.67 (2.55) b,B,2	34.24 (2.62) b,B,1

Same lowercase letters: statistically insignificant differences between materials within the same location/control per heat treatment (same columns). Same uppercase letters: statistically insignificant differences between location/control for the same material per heat treatment (same rows). Same numerical indices: statistically insignificant differences between the same locations/controls within each material between heat-treated and non-heat-treated groups (*p* > 0.05).

**Table 3:** Results of DC% measurements of Tetric EvoCeram for the two spacer sizes and exposure times at central locations along with the directly irradiated controls (means and standard deviations).

Exposure Time	Spacer	
	150 μm	350 μm
**2** × **120 s**	32.07 (4.43) a,A	51.72 (2.03) c,B
**3** × **120 s**	38.13 (2.61) b,A	55.12 (3.14) c,B
	**Controls/preheated**	
**60 s**	51.9 (1.15) d	
**120 s**	61.18 (2.16) e	

Same lowercase letters: statistically insignificant differences per column. Same uppercase letters: statistically insignificant differences per row.

**Table 4:** Results of film thickness measurements (before/after preheating) and the percentage differences vs. the corresponding non-preheated (means and standard deviations). F: Filtek Universal, M: Clearfil Majesty ES-2 Universal, V: Viscalor, T: Tetric EvoCeram.

Materials	Non-Preheated (μm)	Preheated (μm)	Δ[(H-ΝH)/ΝH] %
**F**	423 (11) a,A	355 (29) b,A	−16.1
**M**	261 (23) a,B	210 (14) b,B	−19.5
**T**	202 (12) a,C	164 (21) b,C	−18.8
**V**	237 (13) a,D	158 (19) b,C	−33.3
	**Control (Non-preheated)**		
**E**		104 (11) E	

Same lowercase letters: statistically insignificant differences per materials between heat treatment groups (same rows). Same uppercase letters: statistically insignificant differences between materials per heat treatment and control (same column). Negative Δ values indicate a reduction in film thickness after preheating.

## Data Availability

The original contributions presented in this study are included in the article. Further inquiries can be directed to the corresponding author.
